# A phase 2 study of carfilzomib, cyclophosphamide and dexamethasone as frontline treatment for transplant-eligible MM with high-risk features (SGH-MM1)

**DOI:** 10.1038/s41408-021-00544-x

**Published:** 2021-09-03

**Authors:** Yunxin Chen, Sathish Kumar Gopalakrishnan, Melissa Ooi, Rehena Sultana, Li Hui Lim, Nicholas Grigoropoulos, Shin Yeu Ong, Mingge Xu, SGHMM1 Investigators, Ee Mei Teh, Melinda Tan, Lawrence Ng, Yuh Shan Lee, Colin Phipps, Francesca Lorraine W. Y. Lim, William Hwang, Wee Joo Chng, Yeow Tee Goh, Chandramouli Nagarajan

**Affiliations:** 1 Department of Haematology, Singapore General Hospital, Singapore, Singapore; 2 SingHealth Duke-NUS Blood Cancer Center, Singapore, Singapore; 3 Department of Haematology, Health Sciences North, Sudbury, ON Canada; 4 Department of Haematology, National University Hospital, Singapore, Singapore; 5 Centre for Quantitative Medicine, DUKE-NUS Medical School, Singapore, Singapore; 6 Yong Loo Lin School of Medicine, National University of Singapore, Singapore, Singapore; 7 Department of Clinical Trials & Research Centre, Singapore General Hospital, Singapore, Singapore; 8 Parkway Cancer Centre, Singapore, Singapore

**Keywords:** Myeloma, Cancer therapy

**Dear Editor**,

The incorporation of novel agents in the treatment of MM has improved the median overall survival (OS) from 5 years to ~10 years over the last two decades [1]. However, high-risk MM patients often relapse early with poor OS despite novel therapies [2]. Managing high-risk patients with stem cell transplant, consolidation, and maintenance to achieve deep responses and minimal residual disease (MRD) negativity has been shown to improve survival irrespective of the modality used [3, 4]. Carfilzomib is a potent irreversible proteasome inhibitor that is able to attain deep responses. Carfilzomib has been approved in the relapsed setting having demonstrated its efficacy in the ENDEAVOR and ASPIRE trials [5, 6]. The combination of carfilzomib with cyclophosphamide in the frontline setting has been reported in transplant-ineligible patients [7, 8] and studies are currently ongoing in the transplant-eligible setting [9]. Here, we report results from SGH-MM1, an open-label, phase 2 investigator-initiated study conducted in two tertiary centers in Singapore, which aims to explore the efficacy and safety of carfilzomib, cyclophosphamide, and dexamethasone in the frontline setting for transplant-eligible high-risk MM patients [defined as any of the following: ISS-3, del17p, t(4;14), t(14;16) or 1q21amp)]. No specific cutoffs were used in the definition of del(17p). In the seven patients with del(17p), one patient had del(17p) detected in 13.13% of 200 nuclei, whereas the rest had >50% of nuclei with del(17p). Positivity for 1q21 amplification was defined as any extra copy number of chromosome 1q21 detected on either FISH or karyotyping. In all, 27% of patients had two or more of these high-risk genetic features. This trial was registered at ClinicalTrials.gov as NCT02217163 and approved by the Institutional Review Board.

## Methods

Patients 21 years or older with newly diagnosed MM (NDMM) and high-risk features as defined above, who were transplant-eligible, ECOG 0–2, with adequate organ functions as per protocol were eligible for enrollment. Patients on the study received six 28-day induction cycles of carfilzomib, cyclophosphamide, and dexamethasone (KCyd) comprising of carfilzomib 36 mg/m^2^ on days (D) 1, 2, 8, 9, 15, 16 (carfilzomib was dosed at 20 mg/m^2^ on C1D1 and C1D2), oral cyclophosphamide 500 mg weekly and oral dexamethasone 20 mg twice weekly.

Carfilzomib was initially administered at 56 mg/m^2^/dose according to protocol. After two cases of thrombotic microangiopathy (TMAs) that occurred during the first cycles of induction, the dose of carfilzomib was capped at 36 mg/m^2^ for the rest of the patients after a safety review. Responding patients underwent stem cell collection and proceeded to high-dose melphalan (200 mg/m^2^) and autologous stem cell transplant (ASCT) after six KCyd induction cycles. Following ASCT, patients received two further consolidation cycles of KCyd (doses as per induction). After consolidation, subjects who had at least a Very good Partial Remission (VGPR) had minimal residual disease (MRD) analysis performed through multi-parameter flow cytometry on an adequate bone marrow specimen using a flow analysis protocol adapted from Euroflow with a sensitivity of <1 × 10^−5^ clonal plasma cells defined as MRD-negative [10]. MRD-negative patients were managed expectantly, whereas MRD-positive patients received maintenance carfilzomib 36 mg/m^2^ on D1, 8, 15 every 28 days for 2 years or till disease progression. The primary objective of the study was to determine progression-free survival (PFS) and secondary objectives were overall survival (OS) and MRD status.

Between 29 October 2014 and 11 September 2017, 32 patients were screened and 30 eligible patients received trial treatment. The median age was 61.9 years. Other patient characteristics are shown in Supplementary Table 1. Of the 30 patients, 23 underwent ASCT, and 21 completed consolidation (Supplementary Fig. 1).

**Fig. 1:**
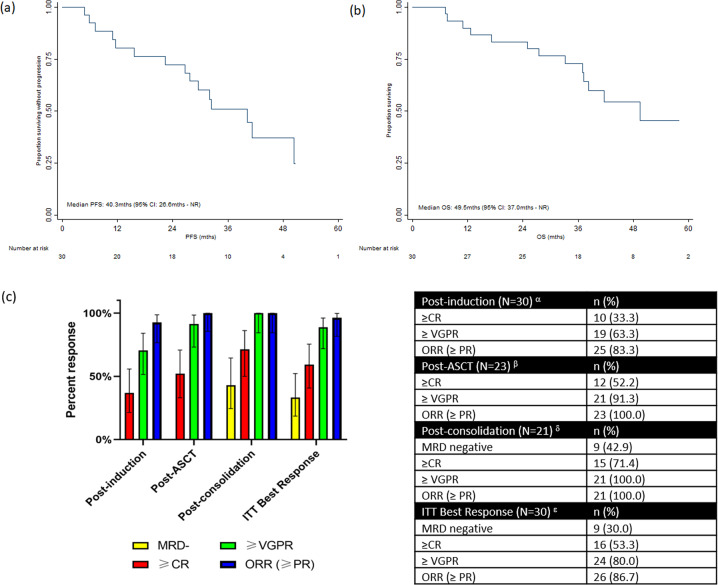
Survival and Response Rates. Kaplan–Meier plot of PFS (**a**), OS (**b**), and response rates (**c**). ^α^Best responses were attained in induction among patients who had received at least one dose of the study drug. ^β^Responses among patients who had completed induction and autologous stem cell transplant. ^δ^Responses among patients who had completed induction, autologous stem cell transplant, and consolidation. ^ε^Best response attained during trial among all patients.

Complete response rates after induction, ASCT, and consolidation were, respectively, achieved by 10/30 (33.3%), 12/23 (52.2%), and 15/21 (71.4%) patients. Respective rates for ≥VGPR was 63.3%, 91.3%, and 100% (Fig. 1). Stem cells were successfully mobilized in all patients attempted, with median (interquartile range) 9.0 × 10^6^/kg (6.3, 12.4) CD34+ cells collected. In all, 21 (70%) of patients completed the planned treatment till consolidation. Of these 9 (42.9%) were MRD-negative and 12 (57.1%) were MRD-positive and received maintenance. Overall MRD negativity rate was 30% (95% CI: 14.7–49.4). Nine patients did not reach the MRD assessment time point (Supplementary Fig. 1). Median PFS for MRD-positive and MRD-negative patients were 41.3 (95% CI: 15.6 mths—not reached (NR)) and 40.3 mths (95% CI: 26.6 mths—NR), respectively.

After a median follow-up of 43.4 months (95% CI: 39.3–55.6 mths), median PFS was 40.3 mths (95% CI: 26.6 mths—NR) and median OS was 49.5 mths (95% CI: 37.0 mths—NR). There was no significant difference in PFS between these two groups (*p* = 0.43).

Safety was assessed in all 30 patients. The average relative dose intensity of carfilzomib (ratio of administered to plan dose) was 82.4% and 86.5% in induction and consolidation cycles. The median dose of carfilzomib delivered in maintenance was 36 mg/m^2^ and the median number of cycles delivered was 20. Hematologic treatment-emergent adverse events (TEAEs) were the most common grade 3/4 events, seen in 13 (43.3%) patients. Other common TEAE were pneumonia, gastrointestinal effects, acute kidney injury, and infective complications. Details are presented in Supplementary Table 2. There were six TEAEs that lead to treatment discontinuation, including three patients with TMA, one patient each with non-ST elevation myocardial infarction (NSTEMI), acute pulmonary oedema (APO), and cytomegalovirus retinitis. Two of the TMAs occurred during induction and the third during the 10th cycle of maintenance with carfilzomib dosed at 36 mg/m^2^. All patients with TMA had complete resolution with cessation of carfilzomib and supportive management without the need for plasmapheresis. These patients were not re-challenged with carfilzomib. The NSTEMI and APO occurred during the first induction cycle and the patients were taken off study. The cardiac events occurred despite baseline adequate cardiac function and judicious management of fluid status. There were no treatment-related deaths in the study.

This phase 2 study demonstrated that KCyd is effective with an acceptable safety profile in transplant-eligible, high-risk NDMM patients. Patients who tolerated and followed through with this regimen had favorable responses with a 71.4% CR rate and 42.9% attaining MRD negativity. Median OS was not reached at a median follow-up of 43.4 months. In the 13 patients who received up to 2 years of carfilzomib maintenance, 2 were discontinued owing to treatment-related AEs and one due to treatment fatigue. The remaining patients tolerated maintenance well.

PIs and immunomodulatory drugs (IMiDs) work synergistically resulting in deep remissions and regimens such as bortezomib, lenalidomide, and dexamethasone, are considered standard of care induction regimens in resource-rich countries [11]. However, this approach is often not achievable in most resource-limited situations. Our study demonstrates that the use of a single potent novel agent, carfilzomib, through induction, consolidation, and maintenance and combining it with a relatively cheaper and efficacious alkylator like cyclophosphamide, can lead to meaningful and durable clinical responses. This approach also makes it possible to treat the first relapse with monoclonal antibody and IMiD-based combinations to bring about durable second remissions.

The role of PI maintenance in the management of high-risk MM patients is increasingly recognized, as reflected in Mayo clinic’s mSMART therapy guidelines. However, long-term use of bortezomib is often limited by therapy-related peripheral neuropathy. Ixazomib, whereas convenient, is a less potent drug in the class. In the phase III Tourmaline MM3 study, the PFS gain for ixazomib over placebo was modest and was considerably less in MRD-positive compared with MRD-negative patients [12]. In our study, carfilzomib maintenance appears to be able to sustain remission for a significant period in MRD-positive patients with survival comparable to MRD-negative patients who did not receive maintenance. A pooled analysis from two phase II European studies in the transplant-ineligible setting showed that carfilzomib was able to mitigate the poor prognosis carried by high-risk cytogenetics, when used in induction and maintenance, with similar PFS and OS compared with standard-risk patients [8].

Carfilzomib at 36 mg/m^2^ was well tolerated suggesting that the early TEAEs were dose-related and this is the optimal dose of carfilzomib when used in KCyd regimen. Other larger studies of this combination by Palumbo et al. and MUK5 trial from the United Kingdom in non-transplant-eligible and relapsed patients and the FORTE study in ASCT eligible patients, all support this finding [9, 13, 14].

Our study has few limitations. The main limitation is the number of patients. High-risk myeloma generally accounts for 15–20% of newly diagnosed patients but increases in proportion in relapsed myeloma patients due to clonal and sub-clonal evolution. It is quite challenging to run a trial for an exclusive HRMM patient cohort for this reason and the risk stratification definition is constantly evolving. For example, 8 of 30 patients had ISS-3 as the only high-risk feature (excluding Del or monosomy 13) in our study and this some might consider as an intermediate rather than a high-risk feature. Our study was conceptualized prior to the publications on R-ISS [2]. At the time, IMWG had published the usefulness of ISS in the risk stratification with and without additional genetic features [15]. Hence, the trial included patients with ISS-3 alone although they were a minority. Further, there was no PFS/OS difference between those with ISS-3 as a single high-risk feature and those with other high-risk features. Another limitation is the use of a single point of MRD measurement and stopping therapy for MRD-negative patients. At the time, the study was commenced not a lot was known regarding MRD, particularly regarding sustained MRD negativity, and trials that had MRD-based treatment stratification were still a novelty. In fact, being able to provide the long-term outcomes of such an MRD-adapted treatment strategy (with the caveat of the single point MRD and a small number of patients) is a motivation for us to present these results. Clearly, our suggestion going forward would be to perform serial MRD measurements to document patients with sustained MRD negativity and to query the ongoing disease evolution as is also recommended by IMWG. Secondly, the routine standard of care in our Institutions at the time of study commencement (and till recently) was induction and consolidation with only very few patients in our practice being able to receive maintenance (due to cost considerations and patient preference). Hence, we considered that giving no maintenance therapy for any given group of myeloma patients as a reflection of our practice and not disadvantaging patients specifically for the purpose of the trial.

KCyd in transplant-eligible NDMM patients is currently being evaluated in the phase 3 FORTE study. FORTE will also provide insights into how KCyd compares to carfilzomib, lenalidomide, and dexamethasone. In the arm where patients received four KCyd cycles followed by MEL200-ASCT and four KCyd consolidation cycles, and thereafter randomized to receive lenalidomide with or without carfilzomib maintenance, ≥CR was attained in 38% of patients pre-maintenance and 41% attained MRD negativity—results that are very similar to our study with the same total number of induction + Consolidation cycles. Notably, the rates of hematological and cardiac toxicities in our study were similar to other KCyd studies with the exception being that 10% of patients developed TMA. We previously detailed a cohort of patients with carfilzomib-related TMAs [16] and postulated concurrent cytotoxic agent, coexisting viral infections, and patient characteristics including ethnicity may have contributed to the higher incidence of carfilzomib-associated TMA. We suggest vigilance for carfilzomib-related TMA with prompt cessation of therapy at occurrence to avert potentially severe consequences.

The demonstration of a good PFS and OS using KCyd supports the notion that upfront strategies using two or more novel agents may not always be necessary for every patient especially when the cost is a key consideration and the relevance of cyclophosphamide even in this era of novel therapies. This may be especially relevant at relapse when more alkylator-naive patients are going to present in the future. Although acknowledging the combination of PI + IMiDS rightly remains the current standard of care, results from our study support continued investigation of KCyd as a more economical and efficacious alternative as a first-line treatment regimen (especially when generic preparations are not available), as compared with regimens such as VRd, in NDMM.

## Supplementary information

Supplementary Figure 1

Supplementary Tables

## References

[1] Fonseca R, Abouzaid S, Bonafede M, Cai Q, Parikh K, Cosler L (2017). Trends in overall survival and costs of multiple myeloma, 2000-14. Leukemia.

[2] Palumbo A, Avet-Loiseau H, Oliva S, Lokhorst HM, Goldschmidt H, Rosinol L (2015). Revised international staging system for multiple myeloma: a report from International Myeloma Working Group. J Clin Oncol.

[3] Jakubowiak AJ, Dytfeld D, Griffith KA, Lebovic D, Vesole DH, Jagannath S (2012). A phase 1/2 study of carfilzomib in combination with lenalidomide and low-dose dexamethasone as a frontline treatment for multiple myeloma. Blood.

[4] Lahuerta JJ, Paiva B, Vidriales MB, Cordón L, Cedena MT, Puig N (2017). Depth of response in multiple myeloma: a pooled analysis of three PETHEMA/GEM Clinical Trials. J Clin Oncol.

[5] Dimopoulos MA, Moreau P, Palumbo A, Joshua D, Pour L, Hájek R (2016). Carfilzomib and dexamethasone versus bortezomib and dexamethasone for patients with relapsed or refractory multiple myeloma (ENDEAVOR): a randomised, phase 3, open-label, multicentre study. Lancet Oncol.

[6] Hari P, Mateos MV, Abonour R, Knop S, Bensinger W, Ludwig H (2017). Efficacy and safety of carfilzomib regimens in multiple myeloma patients relapsing after autologous stem cell transplant: ASPIRE and ENDEAVOR outcomes. Leukemia.

[7] Bringhen S, D’Agostino M, De Paoli L, Montefusco V, Liberati AM, Galieni P (2018). Phase 1/2 study of weekly carfilzomib, cyclophosphamide, dexamethasone in newly diagnosed transplant-ineligible myeloma. Leukemia.

[8] Mina R, Bonello F, Petrucci MT, Liberati AM, Conticello C, Ballanti S (2020). Carfilzomib, cyclophosphamide and dexamethasone for newly diagnosed, high-risk myeloma patients not eligible for transplant: a pooled analysis of two studies. Haematologica.

[9] Gay F, Cerrato C, Rota Scalabrini D, Galli M, Belotti A, Zamagni E (2018). Carfilzomib-lenalidomide-dexamethasone (KRd) induction-autologous transplant (ASCT)-Krd consolidation vs KRd 12 Cycles Vs carfilzomib-cyclophosphamide-dexamethasone (KCd) induction-ASCT-KCd consolidation: analysis of the randomized forte trial in newly diagnosed multiple myeloma (NDMM). Blood.

[10] Flores-Montero J, de Tute R, Paiva B, Perez JJ, Böttcher S, Wind H (2016). Immunophenotype of normal vs. myeloma plasma cells: Toward antibody panel specifications for MRD detection in multiple myeloma. Cytom B Clin Cytom.

[11] Durie BGM, Hoering A, Abidi MH, Rajkumar SV, Epstein J, Kahanic SP (2017). Bortezomib with lenalidomide and dexamethasone versus lenalidomide and dexamethasone alone in patients with newly diagnosed myeloma without intent for immediate autologous stem-cell transplant (SWOG S0777): a randomised, open-label, phase 3 trial. Lancet.

[12] Dimopoulos MA, Gay F, Schjesvold F, Beksac M, Hajek R, Weisel KC (2019). Oral ixazomib maintenance following autologous stem cell transplantation (TOURMALINE-MM3): a double-blind, randomised, placebo-controlled phase 3 trial. Lancet.

[13] Bringhen S, Petrucci MT, Larocca A, Conticello C, Rossi D, Magarotto V (2014). Carfilzomib, cyclophosphamide, and dexamethasone in patients with newly diagnosed multiple myeloma: a multicenter, phase 2 study. Blood.

[14] Maeda T, Wakasawa T, Shima Y, Tsuboi I, Aizawa S, Tamai I (2018). Carfilzomib versus bortezomib in combination with cyclophosphamide and dexamethasone for treatment of first relapse or primary refractory multiple myeloma (MM): outcomes based on genetic risk and long term follow up of the Phase 2 Muk Five Study. Blood.

[15] Chng WJ, Dispenzieri A, Chim CS, Fonseca R, Goldschmidt H, Lentzsch S (2014). IMWG consensus on risk stratification in multiple myeloma. Leukemia.

[16] Chen Y, Ooi M, Lim SF, Lin A, Lee J, Nagarajan C (2016). Thrombotic microangiopathy during carfilzomib use: case series in Singapore. Blood Cancer J.

