# Supplementary figures and images for: A phase 2 study of carfilzomib, cyclophosphamide and dexamethasone as frontline treatment for transplant-eligible MM with high-risk features (SGH-MM1)
Source: Blood Cancer J. 2021 Sep 3;11(9):150. doi: 10.1038/s41408-021-00544-x (PMC8417287; doi:10.1038/s41408-021-00544-x)

Supplementary Figure 1: CONSORT diagram


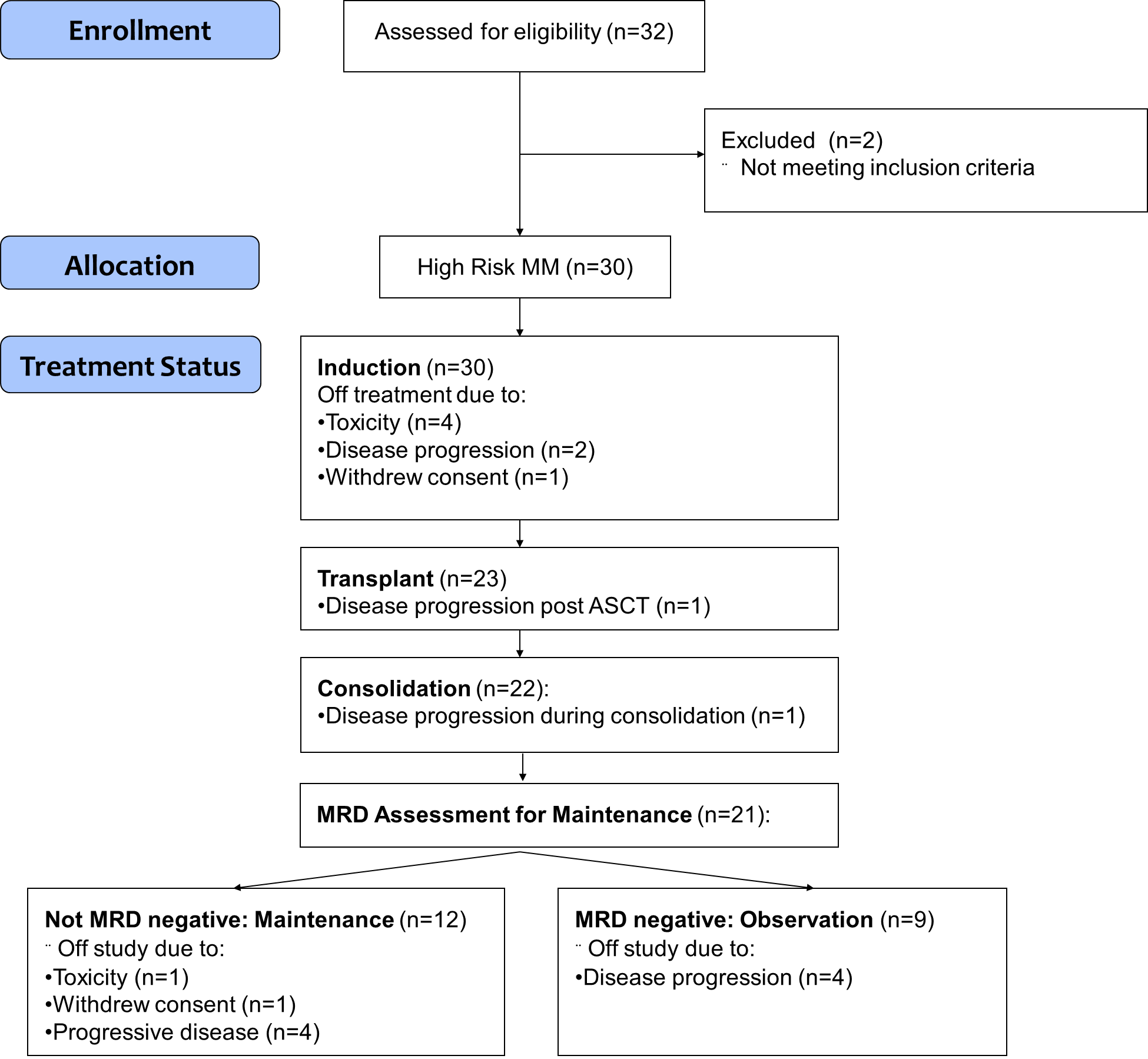

Supplement: Supplementary file 1 — Supplementary Figure 1 [file 41408_2021_544_MOESM1_ESM.docx]
