# Supplementary material for: A phase 2 study of carfilzomib, cyclophosphamide and dexamethasone as frontline treatment for transplant-eligible MM with high-risk features (SGH-MM1)
Source: Blood Cancer J. 2021 Sep 3;11(9):150. doi: 10.1038/s41408-021-00544-x (PMC8417287; doi:10.1038/s41408-021-00544-x)
Supplement: Supplementary file 2 — Supplementary Tables [file 41408_2021_544_MOESM2_ESM.docx]

Supplementary Table 1: Patient Characteristics

| Characteristic |  |
| --- | --- |
| Age  Median - Years (range)  <60 - N (%)  ≥ 60 - N (%) | 61.9 (43-70)  8 (26.7)  22 (73.3) |
| Gender  Male - N (%)  Female - N (%) | 16 (53.3)  14 (46.7) |
| Race  Chinese - N (%)  Malay - N (%)  Indian - N (%)  Others - N (%) | 17 (56.7)  6 (20.0)  6 (20.0)  1 (3.3) |
| Creatinine clearance at diagnosis  <60ml/min - N (%)  60-89ml/min - N (%)  ≥ 90ml/min - N (%) | 18 (60)  8 (26.7)  4 (13.3) |
| R-ISS  I - N (%)  II - N (%)  III - N (%) | 1 (3.3)  16 (53.3)  13 (43.3) |
| Unfavorable FISH / cytogenetics  t(4;14) - N (%)  t(14;16) - N (%)  del17p - N (%)  1q21 amplification - N (%)  ≥ 2 of above features - N (%) | 9 (30)  1 (3.3)  7 (23.3)  12 (40)  8 (26.7) |

Supplementary Table 2: Treatment related adverse events

|  | Any grade  N (%) | Grade 3/4  N (%) |
| --- | --- | --- |
| Treatment Emergent Haematologic Toxicities | | |
| Anemia | 6 (20) | 6 (20) |
| Neutropenia | 11 (36.7) | 7 (23.3) |
| Thrombocytopenia | 7 (23.3) | 4 (13.3) |
| Cardiac | | |
| Acute coronary syndrome | 1 (3.3) | 1 (3.3) |
| Acute pulmonary edema | 1 (3.3) | 1 (3.3) |
| Respiratory | | |
| Pneumonia | 7 (23.3) | 3 (10) |
| Upper respiratory tract infection | 6 (20) | 0 (0) |
| Gastrointestinal | | |
| Diarrhoea | 2 (6.7) | 0 (0) |
| Gastritis | 2 (6.7) | 0 (0) |
| Renal | | |
| Acute kidney injury | 3 (10) | 0 (0) |
| Thrombotic microangiopathy | 3 (10) | 3 (10) |
| Infective | | |
| Bacteraemia | 2 (6.7) | 0 (0) |
| Urinary tract infection | 1 (3.3) | 0 (0) |
| CMV retinitis | 1 (3.3) | 1 (3.3) |
